# Vortical Fountain Flows in Plasticating Screws

**DOI:** 10.3390/polym10080823

**Published:** 2018-07-26

**Authors:** David O. Kazmer, Clemens M. Grosskopf, Varun Venoor

**Affiliations:** 1 Department of Plastics Engineering, University of Massachusetts Lowell, 220 Pawtucket St., Lowell, MA 01854, USA; Varun_Venoor@uml.edu; 2 Fachbereich Maschinenbau und Kunststofftechnik, Hochschule Darmstadt, Gebäude C10, Schöfferstraße 3, Darmstadt 64295, Germany; clemens.m.grosskopf@stud.h-da.de

**Keywords:** single-screw extrusion, fountain flow, residence time distribution, polymer process simulation and modeling

## Abstract

Variances in polymers processed by single-screw extrusion are investigated. While vortical flows are well known in the fluids community and fountain flows are well known to be caused by the frozen layers in injection molding, our empirical evidence and process modeling suggests the presence of vortical fountain flows in the melt channels of plasticating screws adjacent to a slower-moving solids bed. The empirical evidence includes screw freezing experiments with cross-sections of processed high-impact polystyrene (HIPS) blended with varying colorants. Non-isothermal, non-Newtonian process simulations indicate that the underlying causality is increased flow conductance in the melt pool caused by higher temperatures and shear rates in the recirculating melt pool. The results indicate the development of persistent, coiled sheet morphologies in both general purpose and barrier screw designs. The behavior differs significantly from prior melting and plastication models with the net effect of broader residence time distributions. The process models guide potential strategies for the remediation of the processing variances as well as potential opportunities to achieve improved dispersion as well as complex micro and nanostructures in polymer processing.

## 1. Introduction

The global plastics industry exceeds one billion kg in production output per day, with practically all of this material processed by one or more plasticating screws. While extrusion is a steady-state process, it is highly transient in the reference frame of the polymer. In this article, we seek to investigate and model the plastication behavior in the widely used general purpose and barrier screw designs. Our research is guided by modeling and simulation, which support the understanding of the interaction between the machine design, material properties, and processing condition. We are specifically interested in understanding the polymers’ states as a function of position and time within the extrusion screws during plastication.

**Figure 1:**
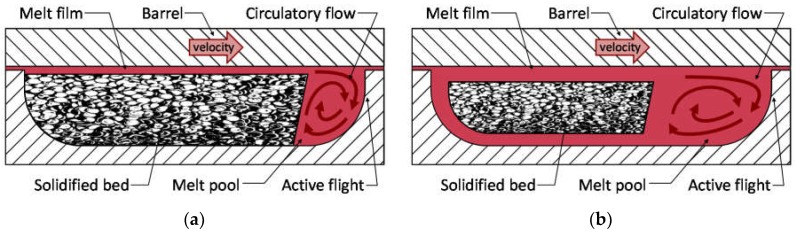
(**a**) Tadmor melting model; (**b**) Five-zone melting model.

Maddock [1] performed screw freezing experiments with single-screw extruders to investigate the melting mechanism of polymer feedstocks, observing that the polymer melt first develops as a film on the surface of the barrel; this behavior was modeled by Tadmor et al. [2,3]. As depicted in the channel cross-section of Figure 1a, the melt film first develops at the end of the feed section of the screw where the significant compression of the polymer provides for improved heat conduction between the polymer and the barrel. The polymer film is then wiped from the barrel by the active flank that is pushing the polymer feedstock down the length of the screw. As the flank flight wipes off the polymer melt film, a melt pool develops adjacent to the active flank. The melt pool is typically the full thickness of the channel adjacent the active flank, with some recirculatory flow. With the further rotation of the screw, the solids bed of polymer feedstock is converted into melt, and the length of the melt pool eventually fills the entire channel.

The five-zone melting model [4] shown in Figure 1b is more representative of the observed behavior, with a higher melting rate than the Tadmor model due to the increased heat transfer and shear stresses applied by the screw surfaces. In particular, the five-zone melting model describes the presence of an outer recirculation zone that assists in the melting and conveying of the solids bed.

**Figure 2:**
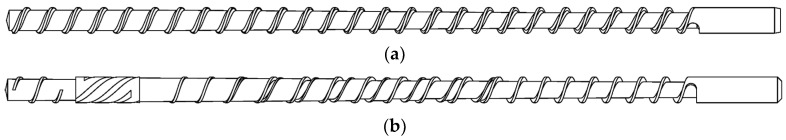
Depiction of two plasticating screws: (**a**) General purpose, square-pitched screw; (**b**) Barrier screw with melt channel, barrier flight, and mixing element.

The historical trend has been to increase processing throughput using longer screws and higher length:diameter (*L*:*D*) ratios. The reason for this trend is that longer screw lengths provide greater residence times and associated heat transfer by viscous dissipation to ensure plastication at higher material flow rates. Screw design guidelines have been established [5,6,7]. The general purpose screw design shown in Figure 2a typically has a pitch equal to the screw diameter for a helix angle of 17.7° and a flight width equal to 10% of the screw diameter. While the channel pitch and depth may vary along the screw axis, design thinking remains dominated by the concept of the compression ratio [8], which is defined as the ratio between the swept volume (proportional to channel width times depth) in the feed section to that of the metering section. The channel depth in the feed section is typically equal to 20% of the screw diameter, decreasing to 10% in the metering section, for a compression ratio of approximately 2:1.

The barrier screw [9,10,11,12] was a significant advance in screw design by incorporating a separate melt channel to ensure complete plastication prior to the metering section. As shown in Figure 2b, the barrier screw design introduces a secondary flight to increase the shearing of the material and associated melting of the pellets. The secondary flight also acts as a “barrier” between the melted polymer and unmelted pellets, thereby avoiding the formation of a melt pool in the primary channel that can act as an insulating layer to prevent pellets from melting efficiently. A full circumferential barrier is often provided at the end of the transition section to guarantee a fully plasticated polymer prior to the mixing section.

## 2. Materials and Methods

### 2.1. Process Investigation

The processing performance of each of the screw designs shown in Figure 2 was characterized. Table 1 provides the screw channel geometry data for the two screws, including the number of flights *n*, the width of the channel *W*, the width of the flight *w*, the depth of the channel *H*, and the rate of change of the channel depth with respect to length *dH/dL*. Each of the screws was designed and built to have a nominal radial clearance of 0.01 mm.

A Davis Standard (Pawcatuck, CT, USA) single-screw extruder with a bore diameter of 38 mm (1.5 in) was instrumented with an auxiliary process controller (MKS SenseLink, Andover, MA, USA) that incorporates real-time computing capabilities with 23 wired analog inputs and 11 wired digital inputs. The instrumentation includes a melt pressure transducer (Gefran M31-6-M, Provaglio d’Iseo, BS, Italy), die pressure transducer (Dynisco PT462E-5M, Franklin, MA, USA), infrared thermocouple (Omega IRT/C-0S36, Norwalk, CT, USA), intrusive melt thermocouple (Tempco-TMB00028, Dale, IL, USA), laser micrometer (Metralight XY, Burlingame, CA, USA), puller velocity (Reliance Minipak-plus, Mumbai, India), system power meter (Acuvim II-M-333-P1, Toronto, ON, Canada), absolute rotary encoder (Koyo TRD-NA1024NWD, Tokyo, Japan), water bath thermocouple, and others.

An extrusion grade of high-impact polystyrene (Dow HIPS Styron 478, Midland, MI, USA) was processed with the melt properties described in Table 2. Both screws were operated at the same process conditions. The barrel profiles for the four zones (from rear to front) were 160 °C, 180 °C, 200 °C and 200 °C. A strand die designed to have an orifice diameter of 3 mm and a land length of 30 mm was also maintained at 200 °C. The screws were operated at 40 RPM to achieve steady-state conditions. Then, a melt flow study was performed in which three 100-g colored charges of HIPS were serially fed into the emptied screw flights. These charges had 5% loadings of black, blue, and violet-colored blends. The colored blends were themselves compounded [13] with a let-down ratio of 25:1 (HIPS to master batch) using a Leistritz twin screw extruder (ZSE 27HP-400, Nuremberg, Germany) with a temperature profile varying linearly from 180 °C at the feed section to 200 °C at the die.

**Figure 3:**
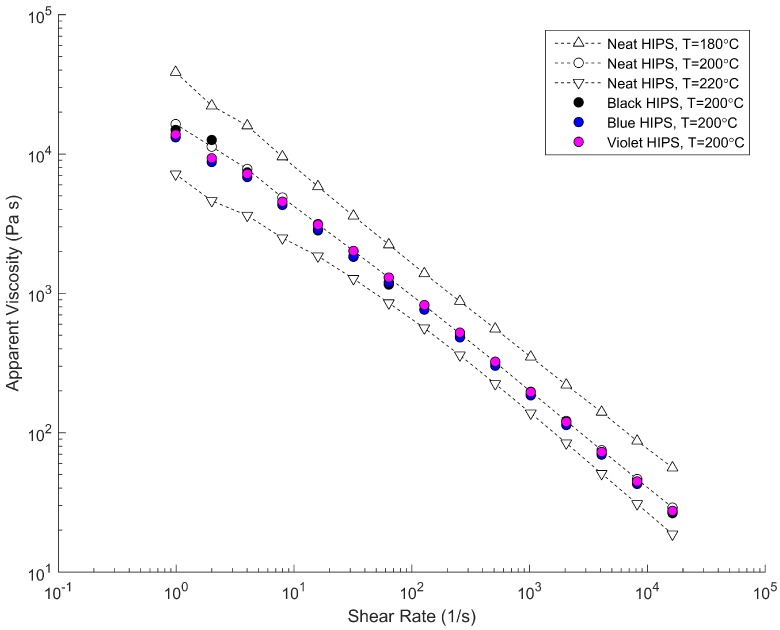
Characterized viscosity data for processed high-impact polystyrene (HIPS 478) and compounded black, blue, and violet blends (CHIPS 1137, CPS 910, CHIPS 1150).

The black, blue and violet blends’ master batches were all polystyrene-based (ECM Plastics’ CHIPS 1137, CPS 910, CHIPS 1150) to ensure compatibility with the HIPS. To investigate the viscosity behavior of the neat HIPS and colored blends, a Dynisco LCR7000 capillary rheometer (Franklin, MA, USA) was used in compliance with ASTM D3835–08 (West Conshohocken, PA, USA). The viscosity at different shear rates and three different temperatures (180 °C, 200 °C and 220 °C) was measured, and is plotted in Figure 3. It is observed that the rheology behavior of the neat HIPS and colored blends are highly consistent.

The extruder was operated at 40 RPM during the addition of the three charges each having 5% of the black, blue, and violet blends. Each charge was added when the prior material being processed fully cleared the feed channel in the first two turns of the screw. Screw freezing experiments (similar to Maddock’s [1]) were then performed by stopping and cooling the extruder after the violet material had cleared the first two turns of the screw. Views of the top and polished cut sections of the frozen processed polymers are subsequently described and analyzed.

### 2.2. Process Simulation

A process simulation was developed to investigate the melting and flow behavior of the polymer during plastication with the general purpose screw. The simulation predicts the flow velocity field **u** as a function of time *t* and space in three dimensions ***x*** by solving the momentum equation [14]:

(1) $$\rho \frac{Du}{Dt}=-\nabla p+\eta \nabla^{2}u,$$

assuming constant density for a non-isothermal, non-Newtonian viscosity. The viscosity is calculated according to the Cross-WLF model [15,16] with the model coefficients provided in Table 2. The temperature field **T** is predicted according to the energy equation including heat conduction, heat convection, and internal viscous heating given the predicted shear stresses and shear rates $\dot{\gamma }$:

(2) $$\rho C_{P}\frac{DΤ}{Dt}+\rho C_{P}\cdot u\cdot Τ=\nabla \cdot k\cdot \nabla Τ+\tau :\dot{\gamma },$$

where the heat capacity **C_P_** and thermal conductivity **k** are modeled as a function of position **x** and temperature **T** according to the linear interpolation of the data in Table 2. The numerical solution was developed to provide high fidelity with respect to flow in the cross-sections of the unwound screw channels by using a semi-implicit finite difference method [17] with 100 layers across the channel width, 100 layers across the channel height, and 34 sections down the channel length corresponding to the half-turns of the last 17 sections of the screw. The solution marched forward for each half-turn of the screw starting at the 11th turn of the general purpose screw. Starting the solution at earlier sections raised numerical stability concerns due to high viscosity predictions at melt temperatures below 105 °C, and also seemed inappropriate in view of the subsequently presented results regarding the modeling of the loosely packed solids bed.

## 3. Results

### 3.1. Observed Behavior

**Figure 4:**
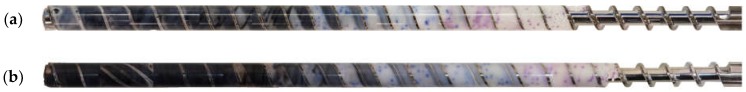
Top views of the frozen-screw pulls: (**a**) General purpose, square-pitched screw; (**b**) Barrier screw with melt channel, barrier flight, and mixing element.

**Figure 5:**
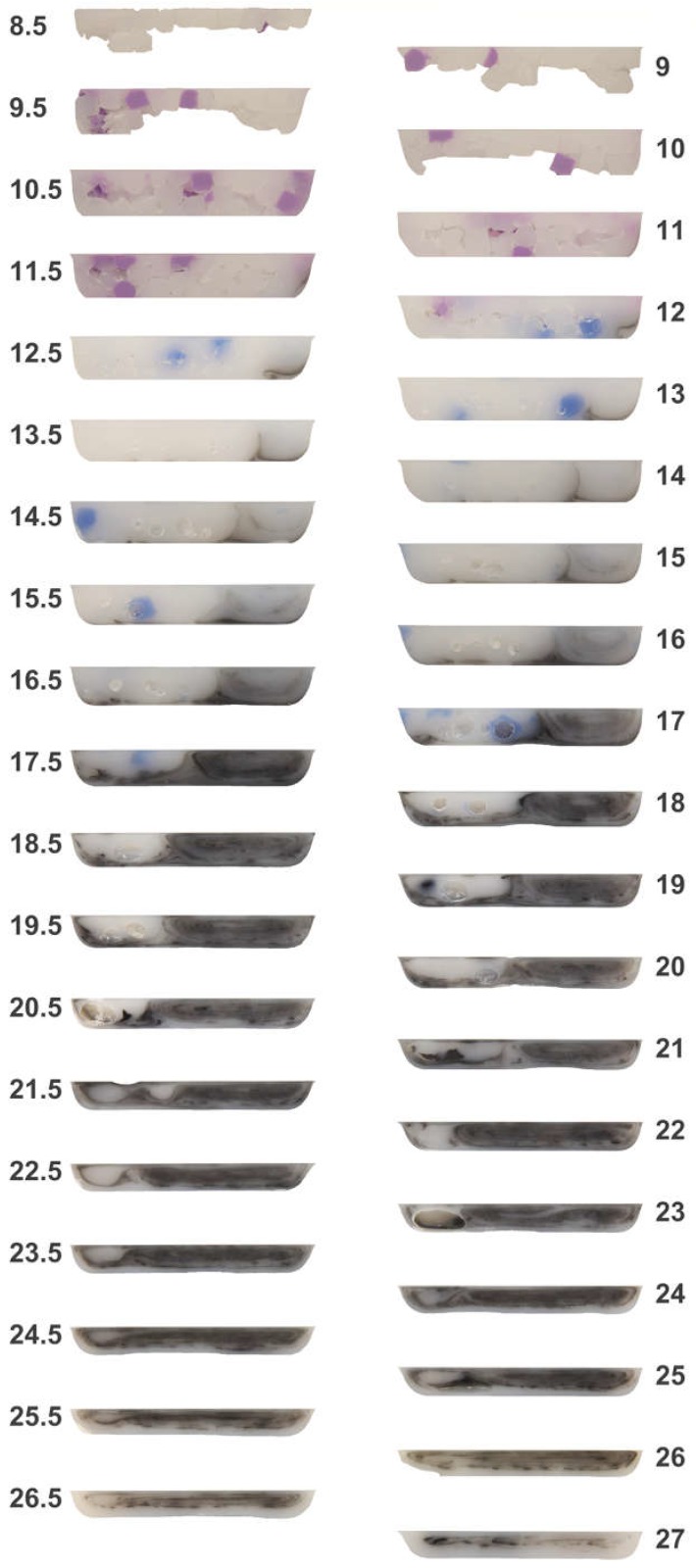
Sections of black, blue, and violet HIPS in the channels of the general purpose screw.

**Figure 6:**
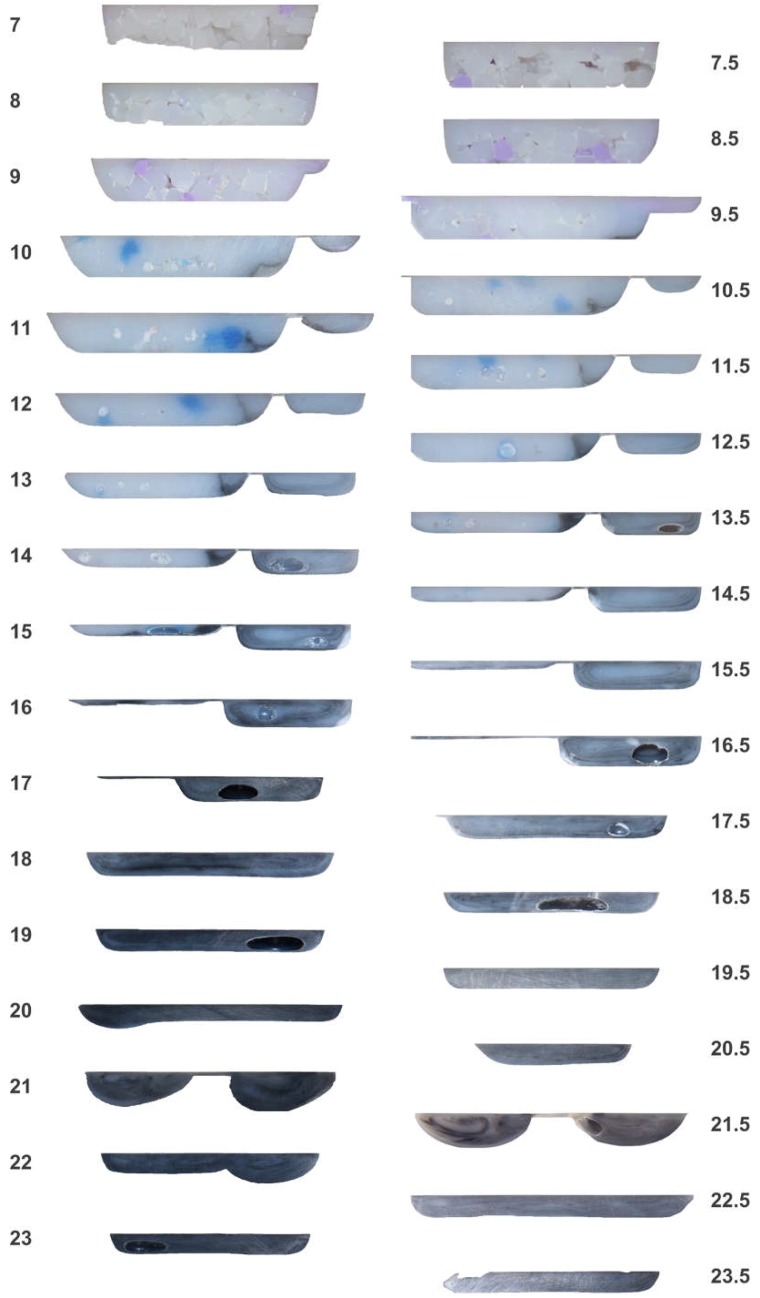
Sections of black, blue, and violet HIPS in the channels of the barrier screw.

Figure 4 provides the top view of the cold screw pulls for the general purpose and barrier screws, wherein an angle of 0° corresponds to the top center of the start of the feed channel. The volume of the 100-g charges of the black, blue, and violet blends were chosen so as to ensure that the black material reached the end of the screw while also ensuring that the violet material entered the start of the transition section. Figure 5 and Figure 6, respectively, provide the imaged cross-sections of the top and bottom channels for the general purpose and barrier screws taken through the plane normal to the top view of Figure 4. The results are significant in many respects, as next discussed.

1. Feed Section Packing: Comparing the external images of the general purpose screw of Figure 5 with the barrier screw of Figure 6, the packed bed of feedstock begins at turn eight, whereas the packed bed in the barrier screw begins at turn seven. Since both screws were operated, stopped, and pulled at the same processing conditions, the variance in the axial start of the solids bed is a significant result. The primary cause is that the general purpose screw has the lowest degree of taper *dH*/*dL* at the end of the feed section. Inspecting rows six through eight of Table 1, it is clear that the barrier screw has three times the rate of change in the channel thickness at the start of the transition section. As such, there is little compression in the feed section of the general purpose screw, and thus little contact pressure to improve heat conduction from the heated barrel to the cooler polymer. The sections for turns 8.5 to 10 in Figure 5 suggest a relatively loosely packed solids bed; the missing pellets below the top surface of these sections fell off upon removal of the sections from the screw channel using a Dremel tool. That the bottom pellets were missing suggests that the pellets nearer the surface of the barrel were hotter, and so were more strongly fused than the pellets closer to the center line of the screw.

The results suggest that the feed section in the general purpose screw could be shortened by at least two turns, or the rate of taper in the transition section increased so as to more efficiently utilize the full length of the screw.

2. Axial Velocity Variation in General Purpose Screw: The varying downstream velocities of the different regions of the channel section are clearly evident from the outer surfaces in Figure 4 as well as the cross-sections of Figure 5 and Figure 6. For example, it is observed from the cross-section at turn 12 in Figure 5 for the general purpose screw that the violet has been conveyed ahead of the blue. At this same turn, we can also observe a significant portion of the black material, which is indicative of a stagnation zone. At turns 12.5 to 17.5, we can also observe the blue material being conveyed ahead of the black material in a recirculation zone.

3. Axial Velocity Variations in the Barrier Screw: Similar velocity variation is also observed in the barrier screw, for example with violet material flowing ahead of the blue material at turn 12, as well as blue material flowing ahead of the black material at turn 20. Indeed, there is even more rapid propagation of the melt stream due to the barrier flight, with the blue blended polymer racing far ahead of the black polymer. The sections of Figure 6 indicate that some of the blue material is conveyed all the way to the front of the screw to turn 22, even while the black material still resides in the solids channel. The underlying reason is that the blue material enters the melt channel early in the transition section, near turn 10, and has a greater axial velocity than the slower moving black material in the solids channel. This axial velocity difference is readily apparent in the external views of Figure 4b, and is indicative of significant differences in the melt residence times in the barrier screw.

4. Tadmor and Shapiro Melting Models Inappropriate for Barrier Screw: Viewing the cross-sections of Figure 6 for the barrier screw, it is evident that the Tadmor and Shapiro melting models are not appropriate for describing the plastication in the solids channel of the barrier screw. The barrier flight, which separates the solids bed from the melt flight, tends to admit the melt film that is in the solids channel adjacent to the barrel into the melt flight. For example, the cross-sections at turns 12 to 15 of Figure 6 show a small stagnation zone toward the right of the solids channel, but no significant division of a recirculation zone and a solids bed as observed for the general purpose screw. The small stagnation zones of the barrier screw suggest that it could be better designed by narrowing the width of the solids channel slightly with the same taper *dH*/*dL*. However, overall, the melting rate in the solids bed of the barrier screw is very impressive compared with that of the general purpose screw.

5. Flow Conductance in Recirculation Zone: The recirculation zones are clearly evident within the general purpose screw, as shown by concentric ovals within the melt pool at the right of the channel (adjacent the active flank) for the cross-sections of turns 14 to 26 in Figure 5. Somewhat surprisingly, the spiral layers in the recirculation zone tend to remain intact, providing the development of a **vortical morphology** [18,19]. Similar recirculations are observed for the melt channel of the barrier screw, as observed in the later cross-sections of Figure 6. The persistence of the vortical morphology is evident, for example, in the melt channel at turns 13–16, after the full barrier at turn 18, and even in the mixing channels at turn 21. This observation is somewhat surprising, since the dispersion provided by the barrier flight is believed to also provide distributive mixing [20].

Regarding the arc length development of the vortex, the tangential velocity of the recirculation zone is much less than the circumferential velocity of the screw relative to the barrel. The cross-section at turn 16 of Figure 6, for example, suggests perhaps six recirculation cycles for an unwrapped length of perhaps 100 mm. This result suggests that there is a significant boundary layer at the surface of the screw that reduces the rotational flow rate in the recirculation zone.

Considering the flow in these recirculation zones, it is recognized that these are areas of relatively high shear, and thus, further viscous heating. The flow conductance is thus higher in the melt channels than the adjacent solids bed, even though they are adjacent in the channel, which explains the previously described axial velocity variation. This axial velocity variation provides a **fountain flow effect** [21,22] whereby material that later enters the feed section via the feed throat can bypass earlier admitted materials currently residing in the screws’ transition sections.

**Figure 7:**
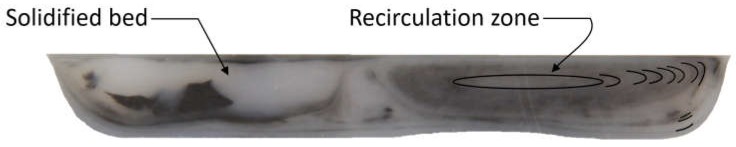
Cross-section of general purpose screw channel at turn 21; the solids bed and recirculation zone having at least eleven recirculations are evident with a poorly worked black pellet in the solids bed.

6. Significant Variances in the Processed Polymer: The varying polymer structures observed in the sections of the general purpose screw may be problematic for at least two reasons. First, there is a very broad residence time distribution since the polymers in the different regions of the channel are flowing at different rates. The black material stagnating early in the screw channel (e.g., turn 12 of both Figure 5 and Figure 6) is also troubling. Second, the significant differences in the flow morphology of the recirculating zone and solids bed are indicative of very different processing histories; the material in the recirculating zone will experience much greater shear flow and thus be at a significantly higher temperature than the material in the solids bed, which remains largely unworked (e.g., the poorly worked black pellet in the solids bed at turn 21 of the general purpose screw in Figure 7).

As the material is pushed off the end of the screw and flows through the die, the differences in the polymer temperature can be expected to cause variances in the melt viscosity that will be observed as differences in the melt pressure, even at constant volumetric output.

### 3.2. Simulation Results

**Figure 8:**
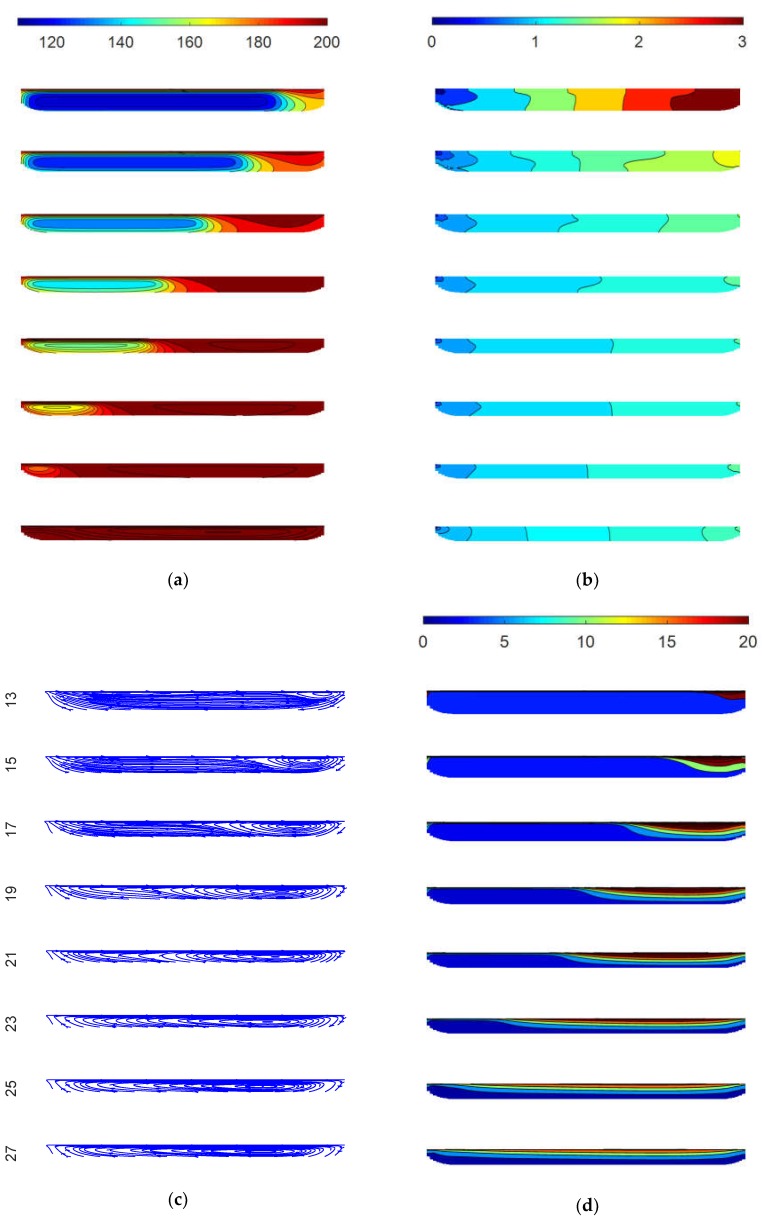
Simulation results for every other turn between 13–27 in the general purpose screw with (**a**) temperature (°C); (**b**) pressure (MPa); (**c**) streamlines; and (**d**) downstream velocity (mm/s).

The simulation results for the cross-section at every other turn of the general purpose screw from turn 13 to turn 27 are shown in Figure 8. Figure 8a provides the temperature distribution, in which the top section corresponds to a large solids bed at an initial temperature of 117 °C. Since this initial temperature is not practically observable, it was derived as a semi-infinite plane with a thickness of 7.6 mm, an initial temperature equal to the feedstock temperature of 30 °C, adjacent walls equal to the set barrel, a screw temperature of 180 °C at this location, and a residence time of 19.5 s derived from the time required for 13 turns of the screw at 40 RPM. Close inspection of the results in the subsequent sections suggests that all of the heat transfer terms (conduction, convection, and viscous heating) are significant. The temperature of the melt pool quickly approaches and exceeds the 200 °C setpoint by turn 19. However, the temperatures in the solids bed are governed primarily by heat conduction, and the temperatures as low as 190 °C persist at turn 25. This temperature difference across the melt channel enables the fountain flow behavior observed in Figure 5. While the final temperature distribution appears uniform in the bottom section, the standard deviation of the predicted temperature is 1.6 °C with a range of 195 °C to 208 °C.

The predicted pressure distributions in the various channels are plotted in Figure 8b. In the top section, the polymer in the channel is at low temperature and presents a very high viscosity. The enforcement of the moving wall at the top of the channel causes a very thin layer of highly shear rates and stresses. For these reasons, the pressure distributions are greatest at the earlier turns with the highest pressures applied by the active flank at the right of the channel. The shear stresses tend to diminish as a function of the channel depth, so the pressure gradient is fairly uniform across the width of the channel, although there are some significant interactions of the pressure with the increased viscosities in the cool solids bed. The pressures are lowest at the final turn shown in the bottom section due to the lower applied shear stresses associated with the polymers’ lower viscosity at this location. The die flow and its associated pressure drop are not modeled, so the outlet pressure is zero.

Figure 8c plots the streamlines at the varying channel sections. The polymer tends to flow toward the right at the top of the channel and toward the left at the bottom of the channel. It is observed that the recirculating flow becomes predominant by turn 15 and subsequently persists. At turns 15 to 23, there is a very slow lateral flow (indicated by the spacing of the leftward directional arrows) through the solids bed due to its high viscosity. The streamlines capture two primary recirculating flows: (1) an inner recirculation corresponding to the vortical flow adjacent to the active flank, and (2) an outer, slower moving recirculation that travels between the bottom of the channel and the solids bed. Both recirculations convey heat to the cooler solids bed, as well as shear stresses that tend to erode the solids bed and cause it to move downstream.

Figure 8d provides the downstream velocity predictions. The polymer travels with a plug flow behavior up to turn 15. Thereafter, the highest velocities occur at turns 15 to 23 of the screw channel. Here, the solids bed’s higher viscosity (and in particular the relatively higher viscosity polymer between the solids bed and the bottom of the screw channel) slows the solids bed velocity. Thus, higher melt downstream velocities occur with vortical fountain flows adjacent to the active flank. In the final turns, the downstream melt velocity is quite consistent across the width of the channel.

## 4. Discussion

**Figure 9:**
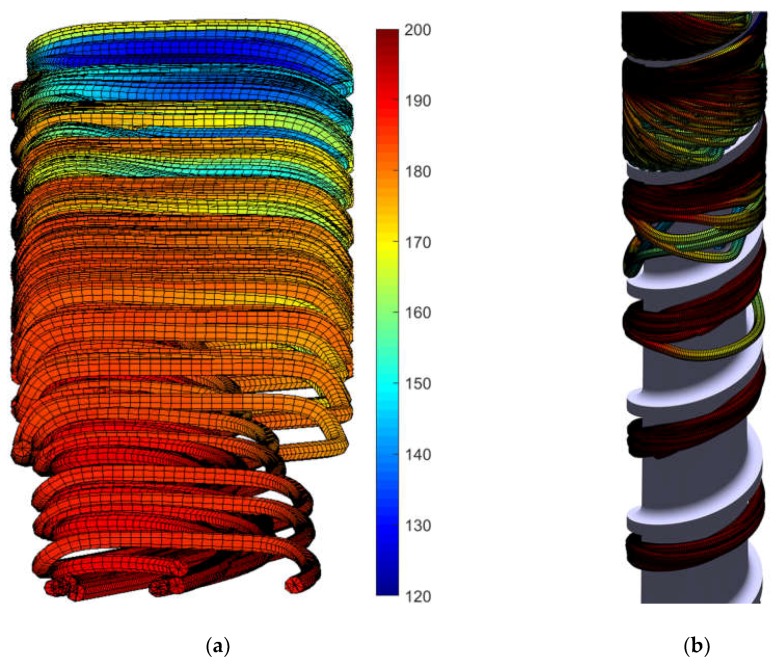
(**a**) Streamlines for melt flows in screw channels starting at turn 13 of the general purpose screw. The length of the streamline is related to the residence time; only eight of the 100 starting particles exit the melt channel after 50,000 iterations with a step size of 0.04 mm; (**b**) Visualized streamlines placed on the screw for turns 18 to 22 with coiling of the recirculating flow in the melt pool.

To assist in visualizing the flow behavior, 100 streamlines starting on a uniform grid at a section of turn 13 were traced and plotted in Figure 9 in which the color is indicative of the instantaneous temperature of the polymer. The length axis is not to scale, and the channel is oriented with the top of the channel facing away from the viewer, and the melt flow progressing from turn 13 at the top of the figure to turn 27 at the bottom of the figure. Many (indeed nearly all) of the 100 streamlines do not exit the cross-section of turn 27. The reason is that the streamline calculation procedure was set to a step size of 0.04 mm and a limit of 50,000 iterations, so that the maximum path length was 2 m.

There are some interesting flow behaviors captured in Figure 9a. In this orientation, the polymer is flowing from left to right along the bottom surface of the channel (facing the viewer) and from right to left along the top surface of the channel (facing away from the viewer). Consistent with Figure 8a, there is a decrease in the melt temperature across the width of the bottom of the channel as the heat is transferred by conduction and convection to the cooler melt in the interior. It can also be observed that there is a significant outer recirculation pattern where the polymer has high flow conductance. The hollow cavities within the streamlines correspond to lower temperature, higher flow resistance zones around which the traveling melt detours.

The internal vortical fountain flow behavior is strongly evident beginning around the center of the model that eventually converges with the outer recirculation zone at the end of the screw channel. Figure 9b plots the same streamlines of Figure 9a rewound about the screw for turns 18 to 22. It is observed that many of the streamlines “stop” in the colder interior of the solids bed. The downstream flow becomes more axial with a coiling of the recirculating flow at the center of the melt pool.

**Figure 10:**
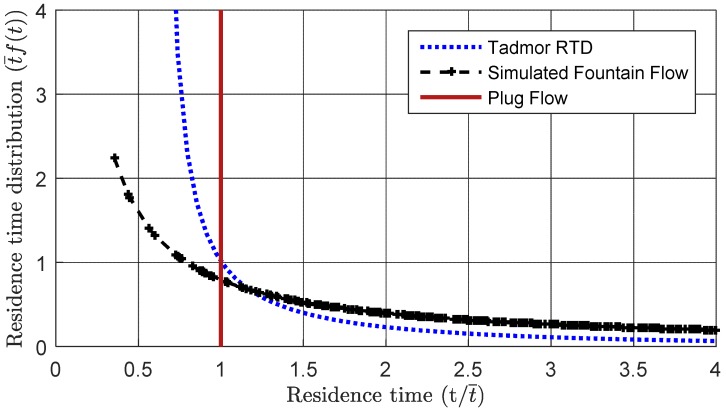
Residence time distribution derived from particle tracking, showing a broader residence time distribution than predicted by Pinto and Tadmor.

The residence time distribution for the presented simulation is plotted in Figure 10 and compared to the theoretical residence time predicted by Pinto and Tadmor [23]. Plug flow would correspond to a vertical line with a constant residence time equal to the mean residence time ($t/\bar{t}=1$). It is interesting to reflect on how the Tadmor RTD model admits some material flowing faster than plug flow. As shown in Figure 10, the effect of the vortical fountain flow is to further accelerate some of the polymer, while the remainder of the polymer being processed will flow more slowly through the other regions of the screw channel. The magnitude of the fountain flow is a function of the screw design, processing conditions, and material properties. The net effect will be to broaden the residence time distribution relative to that modeled by Pinto and Tadmor for extrusion.

The presented results are not without limitations. The presented screw freezing experiments were performed with single screw extruders operated with general purpose and barrier screws under steady-state conditions at 40 RPM. We acknowledge that the extent of the vortical fountain flow will vary with the type of material and operating conditions. Materials possessing greater temperature and shear sensitivity may be expected to exhibit a greater amount of vortical fountain flow, and the effect is expected to increase with higher screw speeds where the processed material will experience more shear heating in the recirculation zone with comparatively less time for temperature equilibration by heat conduction.

Another issues that has been ignored is the role of wall slip at the polymer:barrel and the polymer:screw interfaces. The presented simulation assumed a no-slip condition, which is clearly a significant simplification [24]. Malkin and Patlazhan provide a recent review [25] in which they suggest that wall slip occurs due to liquid-to-solid transitions under shear stress, and is crucially dependent on liquid-to-wall molecular interactions. We believe that wall slip (and its associated surface phenomena) is a critical area of future research to enable model-based polymer processing with respect to accurate modeling of the velocity and temperature field, especially in view of the effective contact velocity and thermal contact resistance.

## 5. Conclusions

The evidence suggests that vortical fountain flows have significant effects in polymer plastication. We believe that the developed coiled sheet morphology could be predicted and exploited to achieve improved dispersion or create micro or nanostructured morphologies within the screw channel. However, higher fidelity material constitutive and processing models are needed to guide researchers and practicing engineers to improve processing capabilities and fully exploit the potential of the processed polymeric systems and composites. For now, variances in temperature and flow rate and pressure will adversely impact most polymer processing applications. To minimize the vortical fountain flow and its adverse effects, practitioners should:

- Minimize the temperature variation between the solids bed and the melt pool by supplying heated polymer feedstock. Such heating will tend to precondition the feedstock and provide improved consistency with a reduced melting length and thus improve melt temperature homogeneity.
- Increase the time for heat conduction to the solids bed by operating the extruder at reduced screw speeds. The reverse (operating the extruder at high speeds) will tend to increase the vortical fountain flow due to less time for heat conduction concurrent with greater shear heating. We now believe that this vortical fountain flow is a predominant cause of the “surging” phenomenon in extrusion and loss of control at higher screw speeds.
- Incorporate additional mixing sections within screw designs. Since the vortical fountain flow is caused by persistently growing melt recirculations in the melt pool adjacent the active flank, intermittently breaking up the melt channel will cause the vortical fountain flows to disperse. All the results, both experimental and theoretical, suggest that the use of early, coarse mixing channels would be highly effective.

## Figures and Tables

**Table 1:** Screw design parameters.

Screw	General Purpose					Barrier				
Turn	*n*	*W*	*w*	*H*	*dH/dL*	*n*	*W*	*w*	*H*	*dH/dL*
1	1	34.29	3.81	7.57	0.0000	1	34.29	3.81	7.57	0.0000
2	1	34.29	3.81	7.57	0.0000	1	34.29	3.81	7.57	0.0000
3	1	34.29	3.81	7.57	0.0000	1	34.29	3.81	7.57	0.0000
4	1	34.29	3.81	7.57	0.0000	1	34.29	3.81	7.57	0.0000
5	1	34.29	3.81	7.57	0.0000	1	34.29	3.81	7.57	0.0000
6	1	34.29	3.81	7.57	0.0000	1	34.29	3.81	7.57	0.0000
7	1	34.29	3.81	7.32	0.0000	2	34.29	3.81	7.57	0.0000
8	1	34.29	3.81	7.06	−0.0020	2	34.29	3.81	7.57	−0.0064
9	1	34.29	3.81	6.81	−0.0020	2	34.29	9.91	6.77	−0.0063
10	1	34.29	3.81	6.55	−0.0020	2	34.29	11.11	5.98	−0.0063
11	1	34.29	3.81	6.30	−0.0020	2	34.29	12.31	5.18	−0.0063
12	1	34.29	3.81	6.05	−0.0020	2	34.29	13.51	4.38	−0.0063
13	1	34.29	3.81	5.79	−0.0020	2	34.29	14.70	3.59	−0.0062
14	1	34.29	3.81	5.54	−0.0020	2	34.29	15.90	2.79	−0.0062
15	1	34.29	3.81	5.28	−0.0020	2	34.29	17.10	1.99	−0.0062
16	1	34.29	3.81	5.03	−0.0020	2	34.29	18.30	1.20	−0.0054
17	1	34.29	3.81	4.78	−0.0020	2	34.29	19.50	0.50	−0.0039
18	1	34.29	3.81	4.52	−0.0020	Not plasticating				
19	1	34.29	3.81	4.27	−0.0020					
20	1	34.29	3.81	4.01	−0.0020					
21	1	34.29	3.81	3.76	0.0000					
22	1	34.29	3.81	3.76	0.0000					
23	1	34.29	3.81	3.76	0.0000					
24	1	34.29	3.81	3.76	0.0000					
25	1	34.29	3.81	3.76	0.0000					
26	1	34.29	3.81	3.76	0.0000					
27	1	34.29	3.81	3.76	0.0000					

**Table 2:** Modeled properties of processed high-impact polystyrene (HIPS) (Styron 478).

Thermal Conductivity		Heat Capacity		Cross-WLF Model (Pa s)	
*T* (°C)	*k* (W/m K)	*T* (°C)	Cp (J/kg K)	Coefficient	Value
38	0.135	51	1511	tau*	10,300
101	0.163	102	2035	a1	25.29
121	0.159	110	2087	a2	51.6
141	0.167	150	2221	d1	1.0 × 10^12^
160	0.164	180	2319	d2	373.15
180	0.169	210	2394	d3	0
199	0.175			n	0.335
218	0.180				
